# Spinal Adhesive Arachnoidopathy, the Disorder More Than Simply Adhesive Arachnoiditis: A Comprehensive Systematic Review of 510 Cases

**DOI:** 10.1111/cns.70084

**Published:** 2024-10-22

**Authors:** Weikang Zhang, Zhenlei Liu, Kai Wang, Lei Zhang, Shaocheng Liu, Xiangyu Zhang, Yutian Wang, Kun He, Hao Wu

**Affiliations:** ^1^ Department of Neurosurgery, Xuanwu Hospital Capital Medical University Beijing China; ^2^ Department of Intensive Care Unit Beijing Mentougou District Hospital Beijing China; ^3^ Department of Neurosurgery Peking University China‐Japan Friendship School of Clinical Medicine Beijing China; ^4^ Department of Neurosurgery China‐Japan Friendship Hospital Beijing China

**Keywords:** adhesive arachnoiditis, arachnoid adhesion, arachnoid cyst, arachnoid fibrosis, arachnoid web, chronic adhesive spinal arachnoidopathy, syringomyelia, systematic review

## Abstract

**Background:**

Spinal adhesive arachnoidopathy (SAA) is a chronic pathology associated with persistent inflammatory responses in the arachnoid. Adhesive arachnoiditis (AA) is one of the major forms of SAA, with accompanying secondary complications. Therefore, we aimed to systematically review both clinical and animal model studies related to SAA to gain a deeper understanding of this unique pathology.

**Methods:**

A literature search was conducted in PubMed, EMBASE, and Cochrane Library databases to retrieve relevant publications up to October 2022. Clinical manifestations, etiologies, imaging modalities, treatments, and prognosis in patients with SAA were collected. Data from animal experiments related to SAA were also extracted.

**Results:**

A total of 176 studies, including 147 clinical and 29 animal model studies, with a total of 510 patients were enrolled in this study. Pain (37.5%), abnormal nerve sensations (39.58%), and abnormal motor function (78.75%) were the top three common symptoms of SAA. Major etiologies included trauma (22.7%), infection (17.73%), surgery (15.37%), and hemorrhage (13.48%). MRI was widely used to confirm the diagnosis. AA could be involved in cervical (96/606, 15.84%), thoracic (297/606, 49.01%), lumbar (174/606, 28.71%), and sacrococcygeal (39/606, 6.44%) vertebral segments. Patients with AA in cervical segments had a higher post‐surgery recovery rate (*p* = 0.016) compared to that of other segments. The common pathological diagnoses of SAA were AA (80.82%), AA combined with arachnoid cyst (12.79%), arachnoid calcification/scars (3.43%), and arachnoid web/fibrosis (2.97%). Patients with AA were more likely to develop syringomyelia, compared with patients with other forms of SAA (*p* < 0.001). Animal studies mainly focused on new AA therapeutic agents (*n* = 14), the pathomechanism of AA (*n* = 14), and the development of new MRI sequences for improved diagnosis (*n* = 1).

**Conclusions:**

The pathological consequences of SAA are more complex than AA and manifest in different forms, such as AA combined with arachnoid cyst, arachnoid calcification/scars, and arachnoid web/fibrosis. In many instances, AA was associated with secondary syringomyelia. Unspecific clinical manifestations of SAA may easily lead to misdiagnosis and missed diagnosis. Although SAA may result from multiple etiologies, including spinal trauma, meningitis, spinal surgery, and hemorrhage, the pathogenesis and treatment of SAA have still not been standardized.

## Graphical abstract

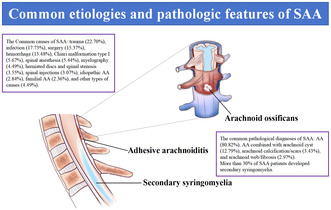

## 1 Introduction

Adhesive arachnoiditis (AA) is generally a rare disease, with less than 1000 cases reported in the past 50 years [1]. The lack of advanced neuroimaging methods in the past was the major roadblock in understanding the clinicopathological nature of AA. Moreover, adhesive arachnoid membranes are frequently encountered during dura mater incisions in spinal surgery and the adhesive membrane is just lysed without further consideration to prevent fibrotic process.

However, AA has been becoming a common pathology in recent years due to a relatively higher incidence of degenerative spinal diseases (DSDs) and an increasing rate of spinal surgery in aged patients [1, 2]. An analysis of multiple publicly available epidemiologic surveys of people with back pain suggests that about 1.75–7 million adult Americans presumably suffer from AA [3, 4, 5, 6, 7, 8, 9]. In our clinical practice, we were initially surprised to observe that even vertebral fusion surgery could not relieve the symptoms in some patients with intervertebral disk herniation and spinal stenosis. However, later these patients were diagnosed with extensive AA. Therefore, nonspecific symptoms (e.g., non‐localized pain, sensory and motor dysfunctions), inadequate medical history presented during outpatient visits, unclarified treatment methods (e.g., steroid injection or lysis surgery), and not following the standard treatment guidelines in AA can lead to high incidences of misdiagnosis and missed diagnosis.

The pathogenesis of AA is still not fully understood. Even the pathological terminology for describing persistent inflammation of the arachnoid membrane and subarachnoid space was not standardized and often inappropriately used as AA, arachnoid webs, arachnoid scars, or arachnoid fibrosis in former studies [10, 11]. In this systematic review, we use the term spinal adhesive arachnoidopathy (SAA) to clarify that AA is just one of the various pathological forms of arachnoiditis. There are also other forms of arachnoidopathy, such as AA combined with arachnoid cyst, arachnoid calcification/scars, and arachnoid web/fibrosis [12, 13]. Moreover, complications of SAA have not been clearly explained in previous literature. Secondary complications such as syringomyelia and arachnoid cysts may also progress toward a more severe or chronic AA pathology [14, 15, 16, 17]. Since AA‐related clinical studies are mostly limited to either case reports or case series and also the number of animal studies on AA is only a few, we aimed to systematically review both clinical and preclinical studies to gain a deeper understanding of SAA.

## 2 Methods

### 2.1 Search Strategy and Study Selection

We searched PubMed Central (PMC), EMBASE, and Cochrane Library for full‐length articles on AA published till October 2022. The medical subject headings and keywords were as follows: “adhesive arachnoiditis OR adhesive leptomeningitis OR arachnoid adhesion OR arachnoid calcification OR arachnoid ossificans.” The full search strategy is presented in Table S1. We included all relevant cases, retrospective studies, and animal model studies related to AA. Articles without full papers or published in other than English languages were also selected if sufficient data were available in the abstract. This review was registered on PROSPERO (registration no.: CRD42023479588).

### 2.2 Data Extraction

We extracted information about the first author name, year of publication, article type, purpose, study characteristics (sample, size, age, gender, group design, conclusion), and clinical characteristics (symptoms, chronic disease, etiology, medical history notes, diagnosis, onset, imaging, pathological location, treatment, prognosis‐mortality, prognosis‐recovery state, follow‐up time).

### 2.3 Statistical Analysis

Statistical Packages of Social Sciences (SPSS) software (version 26.0) was used to analyze the collected data. Parameters with normal distributions were statistically described by means ± standard deviations (SD) and those with non‐normal distributions by medians (interquartile range). The Mann–Whitney *U*‐test was performed to compare the data between the two groups. A chi‐square (χ^2^) test or Fisher's exact test was used to compare data between two or more groups. Any difference in values with *p* < 0.05 was considered statistically significant.

## 3 Results

**FIGURE 1:**
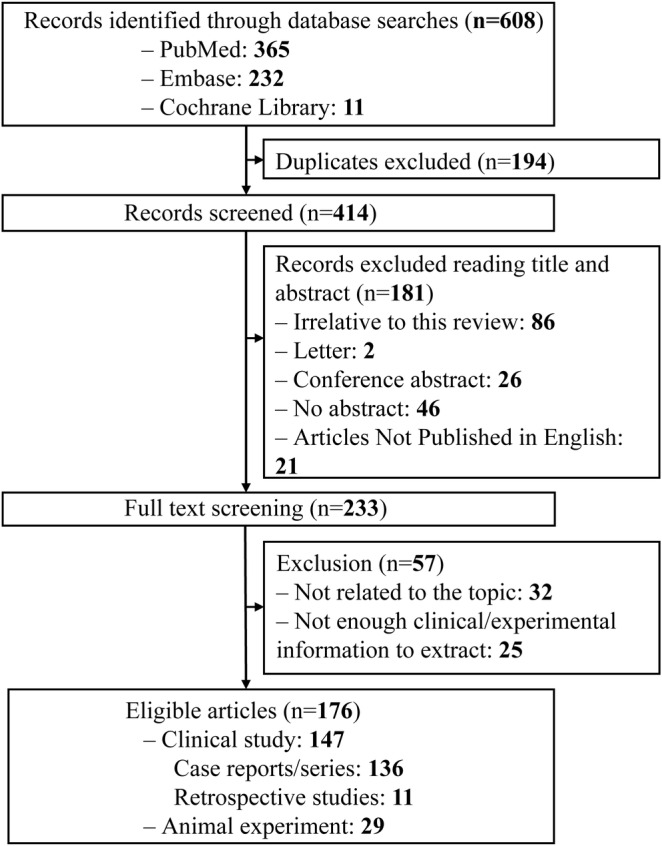
Flow diagram illustrating the study selection process.

We identified a total of 608 publications, and 176 studies were eventually selected for final analysis, which included 147 clinical studies and 29 animal or preclinical studies. The clinical study group consisted of 136 case reports or case series, and 11 retrospective studies involving 510 patients in total. The detailed screening process is shown in Figure 1.

### 3.1 Clinical Manifestation

Patients with SAA usually waited for 12 (3–36) months until the diagnosis was confirmed. Almost 75% of patients suffered from SAA for the first time, while others had recurrent SAA. Cases from Western countries accounted for 62% of the total number of cases, followed by Eastern countries (37%) and African countries (1%). There were 278 males and 232 females, with a mean age of 44.20 ± 17.53 years, in this cohort (Table 1).

**TABLE 1:** Clinical characteristics and risk factors of spinal adhesive arachnoidopathy.

Variables^a^	*n*/*N* (%)
Demographics	
Mean age, years (SD)	44.20 ± 17.53
Gender (male:female)	278:232
Country	
Western	230/371 (61.99%)
Eastern	137/371 (36.93%)
African	4/371 (1.08%)
Time until diagnosis	12 (3–36) months
Onset form	
First time	123/164 (75.00%)
Recurrent	41/164 (25.00%)
Progressive neurological condition	123/277 (44.40%)
Clinical characteristics^b^	
Pain	180/480 (37.50%)
Pain location	
Neck	9/180 (5.00%)
Thoracic	8/180 (4.44%)
Arm	5/180 (2.78%)
Lower back/lumbago	78/180 (43.33%)
Buttocks	4/180 (2.22%)
Saddle region	1/180 (0.56%)
Leg	22/180 (12.22%)
Foot	1/180 (0.56%)
Sciatica	31/180 (17.22%)
Radiating pain	15/180 (8.33%)
Pain types	
Acute	2/16 (12.50%)
Chronic/persistent	2/16 (12.50%)
Intermittent	3/16 (18.75%)
Progressive	9/16 (56.25%)
Abnormal nerve sensations	190/480 (39.58%)
Paresthesia	91/190 (47.89%)
Dysesthesia	39/190 (20.53%)
Hypoesthesia	50/190 (26.32%)
Sensation loss	10/190 (5.26%)
Abnormal motor function	378/480 (78.75%)
Weakness	90/378 (23.81%)
Unilateral upper extremity	6/90 (6.67%)
Bilateral upper extremities	8/90 (8.89%)
Unilateral lower extremity	20/90 (22.22%)
Bilateral lower extremities	41/90 (45.56%)
Paralyzed status	139/378 (36.77%)
Paraparesis	85/139 (61.15%)
Flaccid	4/85 (4.71%)
Spastic	19/85 (22.35%)
Paraplegia/paralysis	21/139 (15.12%)
Tetraparesis	26/139 (18.71%)
Spastic	3/26 (11.54%)
Tetraplegia/tetralysis	5/139 (3.60%)
Gait difficulty	94/378 (24.87%)
Bladder incontinence	92/378 (24.34%)
Feces incontinence	58/378 (15.34%)
Sexual impotence	2/378 (0.53%)
Others	62/480 (12.92%)
Pyramidal syndrome	2/62 (3.23%)
Brown‐Sequard syndrome	2/62 (3.23%)
Medullary syndrome	1/62 (1.61%)
Neck stiffness	4/62 (6.45%)
Dysphagia	2/62 (3.23%)
Dyspnea	2/62 (3.23%)
Decreased/loss of temperature sensation	10/62 (16.13%)
Headaches	16/62 (25.81%)
Nausea/vomiting	3/62 (4.84%)
Muscular atrophy	7/62 (11.29%)
Myelopathy	2/62 (3.23%)
Cauda equina syndrome	5/62 (8.06%)
Tethered cord syndrome	6/62 (9.68%)
Etiologies	
Trauma	96/423 (22.70%)
Infection	75/423 (17.73%)
Meningitis	50/75 (66.67%)
Bacterial meningitis	40/50 (80.00%)
Staphylococcus	1/40 (2.50%)
Listeria monocytogenes meningitis	1/40 (2.50%)
Diplococci meningitis	1/40 (2.50%)
Tuberculous meningitis	31/40 (62.00%)
Viral meningitis	2/50 (4.00%)
Herpes encephalitis	1/2 (50.00%)
Varicella meningoencephalitis	1/2 (50.00%)
Parasitic meningitis	8/50 (16.00%)
Neurocysticercosis	4/8 (50.00%)
Sparganosis	2/8 (25.00%)
Cryptococcal meningoencephalitis	1/8 (12.50%)
Coccidioidomycosis meningitis	1/8 (12.50%)
Spontaneous epidural infection	4/75 (5.33%)
Epidural abscess associated with osteomyelitis	2/75 (2.67%)
Surgery	65/423 (15.37%)
Other surgery	21/65 (32.31%)
Spinal surgery	44/65 (67.69%)
Laminectomy	17/44 (38.64%)
Fusion	5/44 (11.36%)
Spinal tumor resection	2/44 (4.55%)
Foramen magnum decompression	2/44 (4.55%)
Vertebral column resection	1/44 (2.27%)
Hemorrhage	57/423 (13.48%)
Subarachnoid hemorrhage	52/57 (91.23%)
Subdural hemorrhage	4/57 (7.02%)
Epidural hematoma	1/57 (1.75%)
Chiari I malformation	24/423 (5.67%)
Anesthesia	23/423 (5.44%)
Epidural anesthesia	13/23 (56.52%)
Spinal anesthesia	7/23 (30.43%)
Paravertebral anesthesia	3/23 (13.04%)
Myelography	19/423 (4.49%)
Spinal cord stenosis/herniated disk	15/423 (3.55%)
Spinal injections	13/423 (3.07%)
Intrathecal chemotherapy	10/13 (76.92%)
Epidural steroid injection	3/13 (23.08%)
Idiopathic AA	12/423 (2.84%)
Familial AA	10/423 (2.36%)
Others	19/423 (4.49%)
Spinal deformities	6/19 (31.58%)
Kyphosis/lordosis/scoliosis	1/19 (5.26%)
Spinal bifida/myelomeningocele	5/19 (26.32%)
Birth trauma/labor dystocia	4/19 (21.05%)
Traumatic lumbar puncture	3/19 (15.8%)
Autoimmune diseases	5/423 (1.18%)
MCTD	1 (20%)
Polymyalgia rheumatica	1 (20%)
Urticaria pigmentosa	1 (20%)
GBS	1 (20%)
AS	1 (20%)

Abbreviations: AA, Adhesive arachnoiditis; AS, Ankylosing spondylitis; GBS, Guillain‐Barré syndrome; MCTD, Mixed connective tissue disease.

^a^ Categorical variables are given as *n*/*N*, where *n* is the number of patients in which the variable was present, and *N* is the total number of patients for which that particular variable was reported. ^b^The observable and reported traits of a patient's condition.

Patients with AA often presented with pain symptoms in different locations (37.50%), abnormal nerve sensations in related dermatomes (39.58%), and abnormal motor function (78.75%). The primary sites of pain were the lower back (43.33%), leg (12.22%), and sciatica (17.22%). Abnormal nerve sensations in related dermatomes mainly manifested as initial abnormalities such as paresthesia (e.g., numbness and tingling sensations) were experienced by 47.89% of patients but as the disease progressed, more severe conditions such as dysesthesia (e.g., burning and electrical sensations) (20.53%), hyperalgesia (26.32%), or even loss of sensation (5.26%) were observed in these patients. Abnormalities in motor function were manifested by weakness of the limbs (23.81%), gait disturbances (24.87%), and paralysis (36.77%). In lower limbs, bilateral weakness was the most predominant (45.56%), followed by unilateral weakness (22.22%). Unilateral partial paralysis was most common in paralytic patients (61.15%), followed by partial paralysis of the extremities (18.71%). Inflammatory adhesions in the lumbosacral region might involve the cauda equina nerve root, resulting in bladder incontinence (24.34%) and fecal incontinence (15.34%) in SAA patients. A very small number of patients (0.53%) were presented with sexual dysfunctions. Patients with inflammatory adhesions of the arachnoid also reported other clinical manifestations such as headache (25.81%), loss of temperature sensation (16.13%), and muscle atrophy under chronic conditions (11.29%) (Table 1).

### 3.2 Etiology

The common causes of SAA were trauma (22.70%), infection (17.73%), surgery (15.37%), hemorrhage (13.48%), Chiari malformation type I (5.67%), spinal anesthesia (5.44%), myelography (4.49%), herniated disks and spinal stenosis (3.55%), spinal injections (3.07%), idiopathic AA (2.84%), familial AA (2.36%), and other types of causes (4.49%). Surgeries were mainly various types of spinal surgery (67.69%), and infections were most common with meningitis (66.67%), which could be classified as bacterial (80.00%), viral (4.00%), and parasitic meningitis (16.00%). Bacterial meningitis was mostly associated with tuberculosis meningitis, accounting for approximately 62% of cases. There were also some rarer types of infections, including spontaneous epidural infections (5.33%) and epidural abscesses (2.67%). The subarachnoid hemorrhage (SAH) (91.23%) was the most frequent type of hemorrhage. Anesthesia sites were divided into epidural (56.52%), spinal (30.43%), and paravertebral (13.04%), while spinal injection procedures included intrathecal (76.92%) and epidural (23.08%). There were also some rare autoimmune‐related causes (1.18%) that might have contributed to the development of SAA (Table 1).

### 3.3 Imaging Modality and Diagnostic Rate

During the screening stage of SAA, magnetic resonance imaging (MRI) was the most common (79.87%) imaging method due to its noninvasive and relative‐fast scanning time features. Myelograms, X‐rays, and computerized tomography (CT) were only used in 9.96%, 2.88%, and 1.33% of cases, respectively.

To confirm the diagnosis of SAA, some patients even opted for standard invasive methods, such as myelograms (25.00%), diagnostic surgery (4.20%), post‐surgery histopathological examination (1.77%), and fiberscope (1.33%). However, MRI was the mainstream method in 67.48% of cases to confirm the SAA diagnosis. Moreover, high‐resolution and specific MRI sequences particularly designed for detecting cerebrospinal fluid (CSF) obstruction were also introduced to diagnose SAA. A case series of five patients used time‐spatial labeling inversion pulse MRI (T‐SLIP MRI) and another case series of seven patients utilized the three‐dimensional constructive interference in steady‐state MRI (3D‐CISS MRI) for SAA diagnosis. Both studies showed that specially designed MRI sequences could demonstrate CSF obstruction, which was a main pathological feature of SAA (Table 2).

**TABLE 2:** Imaging modalities and diagnostic rate of spinal adhesive arachnoidopathy.

Imaging methods	Screening *n*/*N* (%)^a^	Confirmed diagnosis *n*/*N* (%)^a^
X‐ray	13/452 (2.88%)	0
CT	6/452 (1.33%)	0
MRI	361/452 (79.87%)	305/452 (67.48%)
Myelograms	45/452 (9.96%)	113/452 (25.00%)
Diagnostic surgery	8/452 (1.77%)	19/452 (4.20%)
Histopathological diagnosis	0	8/452 (1.77%)
Fiberscope	6/452 (1.33%)	6/452 (1.33%)
Others	13/452 (2.88%)	1/452 (0.22%)
High‐resolution MRI	1	1
T‐SLIP MRI	5	0
3D‐CISS MRI	7	0

Abbreviations: 3D‐CISS MRI, Three‐dimensional‐constructive interference in steady‐state magnetic resonance imaging; CT, Computed tomography; MRI, Magnetic resonance imaging; T‐SLIP MRI, Time‐spatial labeling inversion pulse magnetic resonance imaging.

^a^ *n* is the number of patients in which the variable was present, and *N* is the total number of patients for which that particular variable was reported.

### 3.4 Subgroup Analysis Based on the Involvement of Different Vertebral Segments

#### 3.4.1 The Lesion Form

In 72.94% of SAA patients, the lesion was located in more than two segments, 20.63% of cases were reported in two segments, and 6.4% of cases had only one segment. The arachnoid membrane in thoracic (49.01%) and lumbar (28.71%) vertebral regions was more likely to be involved in the inflammatory response, compared with that in the cervical (15.84%) and sacrococcygeal (6.44%) vertebral regions. Interestingly, SAA in the cervical region was easily expanded to its adjacent thoracic regions (*p* < 0.001). However, no significant difference was found in the number of inflamed lesions across different subgroups.

#### 3.4.2 Differential Etiology of SAA Involving Different Segments

We selected three common causes of SAA, namely trauma/spinal surgery, meningitis, and SAH. SAA in thoracic vertebral segments was more likely caused by mechanical injury of trauma/spinal surgery (*p* = 0.031), while SAA in cervical segments was more susceptible to inflammatory damages of the meninges (*p* = 0.006).

#### 3.4.3 Post‐Treatment Recovery Rates

Most patients preferred surgery in treating SAA (359/392; 91.6%), while a few cases reported conservative treatments (33/392; 8.4%). The overall post‐surgery recovery rate (84.1%) was significantly higher than that of the post‐conservative treatment recovery rate (51.5%) (*p* < 0.001). Subgroup analysis of different pathological locations showed that SAA patients with lesions in the cervical region had the highest post‐surgery recovery rate compared with that of either thoracic, lumbar, or sacrococcygeal regions (*p* = 0.016). No significant differences in post‐conservative treatment recovery rates were observed among SAA patients with different lesion areas (Table 3).

**TABLE 3:** The lesion forms, etiologies, and prognosis in patients with spinal adhesive arachnoidopathy based on different involved vertebral segments.

Pathological locations	Cervical (*N* = 96)	Thoracic (*N* = 297)	Lumbar (*N* = 174)	Sacrococcygeal (*N* = 39)	All (*N* = 606)	*p*
Lesion forms^1^						
Single vertebral segment	7/96 (7.29%)	21/297 (7.07%)	11/174 (6.32%)	0	39/606 (6.44%)	0.383
Two vertebral segments	15/96 (15.63%)	58/297 (19.53%)	45/174 (25.86%)	7/39 (17.95%)	125/606 (20.63%)	0.189
Multiple vertebral segments	74/96 (77.08%)	218/297 (73.40%)	118/174 (67.81%)	32/39 (82.05%)	442/606 (72.94%)	0.185
Lesions involved in the adjacent region^2^	59/96 (61.46%)^a^	95/297 (31.99%)^b^	39/174 (22.41%)^c^	NA	193/606 (31.85%)	< 0.001
Etiologies^1^						
Trauma/spinal surgery	9/59 (15.25%)^a^	33/95 (34.74%)^b^	11/39 (28.21%)^a,b^	NA	53/193 (27.46%)	0.031
Meningitis	16/59 (27.12%)^a^	15/95 (15.79%)^a^	1/39 (2.56%)^b^	NA	32/193 (16.58%)	0.006
Subarachnoid hemorrhage	8/59 (13.56%)	7/95 (7.37%)	3/39 (7.69%)	NA	18/193 (9.33%)	0.460
Prognosis^1^, ^4^						
Post‐surgery	95.12% (78/82)^a^	84.23% (219/260)^b^	81.62% (111/136)^b^	79.17% (19/24)^b^	84.12% (302/359)^3^	0.016
Post‐conservative treatment	50.00% (4/8)	53.85% (7/13)	45.00% (9/20)	37.50% (3/8)	51.52% (17/33)	0.913

*Note:* a, b, c indicate a significant difference between groups of different pathological locations of adhesive arachnoiditis.

^1^ presented as *n*/*N* (%). *n* is the number of times in which the variable was present, and *N* is the total number of times in which that particular variable was reported. The total amount of included patients was 413; however, some cases reported more than one lesion sites. Therefore, the total amount of reported lesion sites (*N* = 606) was more than that of included patient.

^2^ Extensive adhesive arachnoiditis involved in two adjacent vertebral segments (e.g., cervical and thoracic vertebral segments, thoracic and lumbar vertebral segments, and lumbar and sacrococcygeal vertebral segments).

^3^ The overall post‐surgery recovery rate was significantly higher than the overall post‐conservative treatment recovery rate (*p* < 0.001).

^4^ The prognosis of patients with SAA was evaluated by post‐treatment recovery rate.

### 3.5 Common Pathologic Features of SAA

The common pathological diagnoses of SAA were AA (80.82%), AA combined with arachnoid cyst (12.79%), arachnoid calcification/scars (3.43%), and arachnoid web/fibrosis (2.97%). More than 30% of SAA patients developed secondary syringomyelia. Patients with AA were at higher risk for developing syringomyelia, compared to those with other forms of SAA (*p* < 0.001) (Table 4).

**TABLE 4:** Common pathological forms of the spinal adhesive arachnoidopathy.

SAA	*n*/*N* (%)^a^	Syringomyelia secondary to SAA
Adhesive arachnoiditis	354/441 (80.82%)	139/354 (39.27%)^b^
Arachnoid calcification/scars	15/441 (3.43%)	2/15 (13.33%)
Adhesive arachnoiditis combined with cyst	56/441 (12.79%)	5/56 (8.93%)
Arachnoid web/fibrosis/thickened arachnoid	16/441 (2.97%)	2/16 (12.5%)

Abbreviation: SAA, Spinal adhesive arachnoidopathy.

^a^ *n* is the number of patients in which the variable was present, and *N* is the total number of patients for which that particular variable was reported. *N* = 441.

^b^ Patients with adhesive arachnoiditis showed the highest incidence of secondary syringomyelia among other groups (*p* < 0.001).

### 3.6 Animal Studies on SAA

Animal studies mainly focused on new AA therapeutic agents (*n* = 14), the pathogenesis of AA (*n* = 14), and new MRI sequences for improved diagnosis (*n* = 1). Studies suggest that low concentrations of amitriptyline, the combination of glove powder and pantopaque, piercing at the tattooed site, high doses of radiation, and insufficient decompression after arachnoid resection are likely to increase the risk of adhesions [18, 19, 20, 21, 22]. The peripheral benzodiazepine receptor agonist (Ro5‐4864), tamoxifen (TAM), recombinant tissue plasminogen activator (rt‐PA), mitomycin C, and pimecrolimus significantly reduced the degree of epidural fibrosis as well as the risk of adhesion [23, 24, 25, 26]. Two randomized trials conclude that low doses of radiation inhibit the development of epidural fibrosis, while high doses may increase the probability of fibrosis occurring [19, 27]. Four trials show that epidural substitutes such as HA tablets, polyvinyl alcohol hydrogel tablets, Gore‐Tex films, and DuraGen can exert potential anti‐inflammatory effects and could be effective in preventing the development of adhesions and scarring [28, 29, 30, 31]. Furthermore, two trials suggest that lavage is effective in reducing the incidence of arachnoid adhesions after meningitis [32, 33]. Brain pool lavage reduced the incidence of arachnoid adhesions after meningitis, and epidural microbial inhibitor lavage has been proven effective in preventing the formation of adhesions and fibrosis. Subarachnoid shunt and adhesion lysis are effective surgical methods for the treatment of SAA. Besides, if accompanied by disk herniation, SAA can also be treated by lateral corpectomy [34, 35]. Five studies explored the pathomechanism of CSF circulation disorder caused by post‐traumatic inflammatory responses and adhesions, leading to intramedullary edema and syringomyelia [36, 37, 38, 39, 40]. One study reported the generation of an animal model mimicking the formation of post‐traumatic spinal cord cavities, resulting in post‐traumatic syrinxes and arachnoid cysts through intracerebral injection of quisqualic acid (QA) and kaolin into the parenchyma [41]. Another study showed that commercially available methylprednisolone could increase the incidence of SAA due to the presence of polyethylene glycol (PEG) in its formulation [42] (Table 5).

**TABLE 5:** Experimental studies related to spinal adhesive arachnoidopathy.

References	Animal model	Sample size	Study purpose	Study design/intervention	Main findings^a^
Keskin, E et al. 2021	Post‐laminectomy (Rat)	32	Exploration of the histopathological effects of Ro5‐4864 on EF	C: No treatment after surgery (*n* = 8) I1: Hemostasis after surgery through an absorbable gelatin sponge (*n* = 8) I2: Low‐dose (4 mg/kg) Ro5‐4864 for 30 min before the surgery (*n* = 8) I3: High‐dose (8 mg/kg) Ro5‐4864 for 30 min before the surgery (*n* = 8)	The Ro5‐4864 could be effective in reducing EF in rats
Da Silva, R. A et al. 2019	Tattooed skin (Rabbit)	42	Evaluation of the inflammatory response induced by spinal puncture through tattooed skin in the meninges, spinal cord, and arachnoid	C: Spinal puncture through non‐tattooed skin and saline solution injection (*n* = 14) I1: Spinal puncture through tattooed skin and saline solution injection, with shorter observation time (30 days) (*n* = 14) I2: Spinal puncture through tattooed skin and saline solution injection, with longer observation time (360 days) (*n* = 14)	Spinal puncture through the tattooed skin of rabbits can trigger acute inflammatory changes in the meninges, which would evolve into adhesive arachnoiditis after long observation
Tauro, A. 2018	SAD (Dog)	38	Application of 3D‐CISS sequences in routine MRI to diagnose SAD or AA	C: MRI with 3D‐CISS sequences in healthy dogs (*n* = 19) I1: MRI with 3D‐CISS sequences in SAD dogs (*n* = 19)	The 3D‐CISS sequence could delineate SAD from other changes associated with abnormal CSF hydrodynamics and provide more anatomical details than conventional MRI sequences
Ozturk, Y. 2018	Post‐laminectomy (Rat)	24	Exploration of the effectiveness of TAM in AA and fibrosis treatment	C: No treatment post‐laminectomy (*n* = 8) I1: Spongostan in the operation lodge during laminectomy (*n* = 8) I2: TAM post‐laminectomy (*n* = 8)	TAM may reduce EF, dural thickness, and inflammatory response after laminectomy in rats
Meren, I. L. 2017	CSF flow obstruction (Dog)	7	Description of a novel technique for ameliorating cerebrospinal fluid flow obstruction secondary to pia‐arachnoid fibrosis	Subarachnoid‐subarachnoid shunt via shunt tube for the amelioration of cerebrospinal fluid flow obstruction caused by spinal cord subarachnoid fibrosis (*n* = 7)	Bridging a region of pia‐arachnoid fibrosis with a tube placed in the subarachnoid space can ameliorate or prevent the progression of associated clinical signs
Yokogawa, N. 2015	Radiotherapy (Mice)	8	Histopathologic assessment of post‐irradiation changes in the spinal dura mater and peridural tissue in mice	C: No irradiation after surgery (*n* = 3) I1: Radiotherapy (10 or 20 Gy at 150 kV, 20 mA) to the thoracolumbar transition after surgery (*n* = 5)	Spinal EF develops in the late stages after high‐dose irradiation, and it may be associated with overexpression of TGF‐β1. Additionally, thinning of the arachnoid barrier cell layer was observed in the late stages after irradiation
Bismuth, C. 2014	Chondrodystrophy (5 Pugs and 1 French Bulldog)	6	Description of features of thoracolumbar spinal subarachnoid cysts and assessment of post‐surgery outcomes	Removal of spinal subarachnoid cysts (*n* = 6)	The thoracolumbar subarachnoid cysts were associated with leptomeningeal adhesions. Good results seemed to be obtained by dissecting and removing these adhesions. Spinal cord compression may be associated with disk protrusion, particularly in chondrodystrophic dogs, and protruding disk can be treated by lateral corpectomy
Kobayashi, S. 2012	SAA (Rabbit)	30	Investigation of the blood permeability and ultrastructural changes of the spinal cord vessels in spinical AA	C: No kaolin into the subarachnoid space at the T8 (*n* = 15) I: Kaolin into the subarachnoid space at the T8 (*n* = 15)	The disturbance of CSF flow associated with circumferential obstruction of the subarachnoid space appears to be closely related to the onset of hydromyelia. The onset of syringomyelia seems to be closely linked to the breakdown of the blood–spinal cord barrier caused by disturbed intramedullary blood flow, particularly venous congestion, and the intramedullary edema that occurs as a result
Lima, R. M. 2010	Injection of methylprednisolone (Dog)	14	Assessment of the clinical and histological changes associated with the injection of methylprednisolone into the intrathecal space of dogs	C: 1 mL of 0.9% normal saline into the intrathecal space (*n* = 7) I: 1 mL (1.15 mg/kg) of methylprednisolone into the intrathecal space (*n* = 7)	The intrathecal administration of commercially available methylprednisolone was responsible for causing histological changes in the spinal cord and meninges of the animals studied
Fukushima, F. B. 2009	Injection of amitriptyline (Dog)	21	Assessment of the clinical and histological toxicity of intraspinal amitriptyline at the lowest dosages previously known to be effective	C: 1 mL of 0.9% saline into the intraspinal space (*n* = 7) I1: 1 mL of 0.15% amitriptyline into the intraspinal space (*n* = 7) I2: 1 mL of 0.3% amitriptyline into the intraspinal space (*n* = 7)	The intraspinal administration of amitriptyline to dogs even in low concentrations is strongly associated with the development of intense meningeal AA and is not safe even at low concentrations
Cemil, B. 2009	Post‐laminectomy (Rat)	30	Investigation of the preventive effects of the local application of pimecrolimus in minimizing spinal epidural fibrosis	C: No treatment after surgery (*n* = 10) I1: A cotton pad soaked with MMC (0.5 mg/mL) on the exposed dura mater (*n* = 10) I2: A cotton pad soaked with 5 mg pimecrolimus on the exposed dura mater (*n* = 10)	The application of local pimecrolimus effectively reduces epidural fibrosis and dural adherence in rats that underwent lumbar laminectomy. MMC was equally effective as pimecrolimus in reducing epidural fibrosis and dural adherence
Seki, T. 2008	PTS (Rat)	64	Description of greater insight into the mechanisms of this degenerative sequela of SCI	C: No treatment after surgery (*n* = 6) I1: 35 g clip compression injury after surgery (*n* = 21) I2: 35 g clip compression injury +5 μL of 500 mg/mL kaolin solution after surgery (*n* = 22) I3: 5 μL of 500 mg/mL kaolin without after surgery (*n* = 15)	The combination of compressive/contusive SCI with induced arachnoiditis results in severe PTS and perilesional myelomalacia, which is associated with enhanced inflammation, astrogliosis, and apoptotic cell death
Kato, T. 2005	Post‐laminectomy (Rabbit)	23	Determination of the efficiency of HA sheet for the prevention of postlaminectomy adhesions compared with that of HA gel or another sheet	C: Nothing over the defect (*n* = 5) I1: A GF sheet over the defect (*n* = 9) I2: A HA sheet over the defect (*n* = 9)	In an experimental laminectomy model, the HA sheet formed a solid interposition membrane barrier and exhibited anti‐inflammatory activity
Haq, I. 2005	Post‐laminectomy (Pig)	26	Evaluation of the degree of chronic inflammatory reactions, adhesions, and fibrosis caused by the use of four dural substitutes—Surgicel, Durasis, DuraGen, and Preclude	I1: Surgicel in the subarachnoid space (*n* = 5) I2: Durasis in the subarachnoid space (*n* = 5) I3: DuraGen in the subarachnoid space (*n* = 5) I4: Preclude in the subarachnoid space (*n* = 5) Six animals were excluded from the study	The DuraGen dural substitute produced the least amount of inflammation in the subarachnoid space and Preclude generated the most. Surgicel and DuraGen both produced some inflammation, which diminished gradually from the dural implant center. Durasis caused the least degree of inflammatory cell infiltration. The Preclude dural substitute consistently demonstrated encapsulation and arachnoidal reaction
Aydin, M. D. 2005	Meningitis (Lamb)	12	Investigation of the role of cisternal irrigation in preventing meningitis complications	C: No treatment (*n* = 2) I1: The cefotaxime (4 × 1 g/day) for 20 days (*n* = 5) I2: The cefotaxime (4 × 1 g/day) for 20 days + cisternal irrigation (*n* = 5)	Meningitis can affect all central neural tissues, which may result in serious central nervous system lesions. The irrigation procedure may decrease the percentage and severity of meningitis complications by excreting inflamed purulent collection from the subarachnoid spaces
Gnirs, K. 2003	Spinal arachnoid enlargement (Dog)	13	Exploration of spinal subarachnoid cyst pathogenesis	Preoperative myelography (*n* = 13) Dorsolateral hemilaminectomy to detect cysts (*n* = 12) Examination of the pathologic specimens (*n* = 5); cross‐sectional CT scanning (*n* = 5); multiple‐sequence MRI (*n* = 1)	Adhesive arachnoiditis secondary to various inflammatory processes maybe a potential cause for subarachnoid cyst formation
Kemaloglu, S. 2003	Post‐laminectomy (Rat)	24	Investigation of the effectiveness of rt‐PA on post‐laminectomy epidural fibrosis in rats	C: No treatment after surgery (*n* = 12) I1: Rt‐PA was inserted on the dura mater after surgery (*n* = 12)	The topical thrombolysis with rt‐PA is safe and efficacious in preventing post‐laminectomy EF and arachnoiditis
Brodbelt, A. R. 2003	Syringomyelia (Rat)	92	Improvement of the model to produce syrinx in animals, to examine the relative influences of excitotoxic injury and neuronal loss on syrinx formation	C1: The examination of MRI (*n* = 6) C2: Single intraparenchymal injection of Evans blue after surgery (*n* = 4) C3: Subarachnoid kaolin after surgery (*n* = 4) I1: Single intraparenchymal injection of QA + subarachnoid kaolin (*n* = 46) I2: Multiple intraparenchymal injection of QA + subarachnoid kaolin (*n* = 20) Not in the remainder of the study (*n* = 12)	QA causes rapid non‐progressive neuronal loss and appears responsible for an initial cyst formation. Increased QA dose increases the size and rate of syrinx formation, suggesting the formation of an initial cyst may be an important part of syrinx pathogenesis. Active fluid flow is responsible for the enlargement of the initial cyst
Bora, H. 2001	Post‐laminectomy (Rabbit)	30	Exploration of whether the radiotherapy could inhibit peridural fibrosis after laminectomy and the efficiency of external irradiation was compared with spinal membrane application	C: No treatment after surgery (*n* = 10) I1: Radiotherapy (a single fraction of 900‐cGy external irradiation administered by 9‐MeV electron beam 24 h) after surgery (*n* = 10) I2: Application of spinal membrane during surgery (*n* = 10)	This preliminary study showed that high‐single‐fraction/low‐total‐dose administered postoperatively can successfully inhibit postsurgical epidural fibrosis as effectively as applied spinal membrane
Miaki, K. 1999	Post‐laminectomy (Rat)	44	Exploration of how the nutritional supply to the cauda equina is changed in lumbar adhesive arachnoiditis	C: No operation (*n* = 12) I1: L5‐L6 laminectomy (*n* = 12) I2: Kaolin (5 mg) into the dorsal extradural space post L5‐L6 laminectomy (*n* = 20)	The glucose transport to the cauda equina from the vessels increased by 53% and that from the cerebrospinal fluid remarkably decreased by 72% compared with the normal cauda equina. The impairment of nutritional supply to adhered cauda equina may lead to eventual neural degeneration
Park, Y. K. 1998	Post‐laminectomy (Rat)	72	Evaluation of the arachnoidal adhesions and inflammation after spinal dural repair with one of three materials: Gore‐Tex surgical membrane, collagen‐coated Vicryl mesh, or lyophilized spinal dural allograft	I1: A Gore‐Tex surgical membrane in the subarachnoid space (*n* = 24) I2: A Collagen‐coated Vicry lmesh in the subarachnoid space (*n* = 24) I3: A Lyophilized dural allograft in the subarachnoid space (*n* = 24)	The Gore‐Tex surgical membrane was a very good material for the surgical repair of spinal dural defects. Additional experimental studies are required to com pare Gore‐Tex membrane with autologous tissues
Transfeldt, E. E. 1995	Post‐laminectomy (Cat)	30	Investigation of the usefulness of a polyvinyl alcohol hydrogel sheet as an interposition over the dura to prevent inflammatory and adhesive reactions post‐laminectomy	C: No treatment after surgery (*n* = 10) I1: A polyvinylalcohol hydrogel sheet over the defect (*n* = 10) I2: The free fat graft over the defect (*n* = 10)	The polyvinyl alcohol hydrogel sheet prevents the migration of inflammatory cells and subsequently reduces intraspinal canal scar tissue formation and adhesive reaction. Other beneficial properties are extreme elasticity and low friction. The polyvinyl alcohol hydrogel sheet is believed to be useful in eliminating scar tissue formation and does not interfere with the dynamic gliding movement of the spinal cord and nerve roots
Cho, K. H. 1994	Syringomyelia (Rat)	38	Elucidation of the role of spinal blockade in posttraumatic syringomyelia	C: Traumatic injury (*n* = 8) I1: Traumatic injury +100 mg kaolin suspended in 1 cc normal saline solution into the subarachnoid space (*n* = 12) I2: Traumatic injury +200 mg kaolin in 1 cc normal saline solution into the subarachnoid space (*n* = 9) I3: No traumatic injury +200 mg kaolin in 1 cc normal saline solution into the subarachnoid space (*n* = 9)	The difference between the frequency of syrinx formation and the time of survival was statistically significant. Subarachnoid block secondary to adhesive arachnoiditis is important in initiating the extension of the syringomyelia cavity
Reigel, D. H. 1993	Laminectomy and surgical meningeal injury (Rabbit)	12	Evaluation of the potential of P407 to prevent the production of arachnoid adhesions and nerve root scarring post laminectomy and surgical meningeal injury	C: No P407 at the level of the spinal cord (*n* = 4) I1: P407 at the level of the spinal cord (*n* = 8)	There was a 50% reduction in leptomeningeal adhesion formation with the use of P407. P407 may be useful in neurosurgery for the prevention of arachnoid adhesions
Strohecker, J. 1985	Lumbar laminectomy (Rabbit)	23	Evaluation of the effect of the microbicid Polyvinylpyrrolidon‐Jod‐Komplex as a lavage in epidural space	C: No operation (*n* = 3) I1: P.J.K. diluted 1:1 with physiological saline as lavage during surgery (*n* = 10) I2: Sterile physiological saline during surgery (*n* = 10)	Local epidural P.J.K.‐lavage is harmless and might be beneficial in the prevention of perioperative infections and in neurosurgery without risk of arachnoiditis or increased scar formation
Oiwa, T. 1985	Posterior cervical laminectomy (Dog)	40	Exploration of postoperative changes around the dural meter at the site of cervical laminectomy, which is influenced by intradural operative procedures and the flow of cerebrospinal fluid	C: Sham surgery at C6 level (*n* = 9) I1: Cervical laminectomy with incomplete decompression at C6 level (*n* = 9) I2: Cervical laminectomy with incomplete decompression at C4 and C6 levels (*n* = 17) I3: Cervical laminectomy with complete decompression at C4–C6 levels (*n* = 5)	The scar tissue did not adhere to the spinal cord after arachnoid resection in normal dogs, adhesion of scar to the spinal cord was seen to a small extent after arachnoid resection with sufficient posterior decompression in dogs whose spinal cord was compressed anteriorly by a screw through the vertebral body. In the cases with insufficient posterior decompression, adherence was observed much more extensively
Williams, A. G. 1982	Healthy New Zealand white rabbit	51	Exploration of the effects of surgical glove powder contamination in the cerebrospinal fluid	C: Suboccipital puncture only (*n* = 6) I1: High dose: 0.1 mL Pantopaque (*n* = 9) 10 mg Biosorb in 0.1 mL NaCI (*n* = 10) 10 mL Biosorb in 0.1 mL Pantopaque (*n* = 10) I2: Low dose: 100 μg Biosorb in 0 0.1 mL NaCI (*n* = 8) 100 μg Biosorb in 0 0.1 mL Pantopaque (*n* = 8)	The combination of glove powder and pantopaque is synergistic in producing arachnoiditis
Wagner Jr., F. C. 1977	Post‐Laminectomy (Cat)	40	Delineation of the pathological changes occurring in the spinal cords of experimental animals from 4 h to 4 months after graded trauma based on different weights	I1: Transdural trauma from 100 g of weight at a specific height after surgery (*n* = 10) I2: Transdural trauma from 300 g of weight at a specific height after surgery (*n* = 10) I3: Transdural trauma from 500 g of weight at a specific height after surgery (*n* = 10) I4: Transdural trauma from 700 g of weight at a specific height after surgery (*n* = 10)	Localized intramedullary cavitation will develop following nondisruptive spinal cord trauma if the magnitude of the original trauma and resulting vascular damage is sufficient. Although adhesive arachnoiditis also occurs with similar amounts of trauma, the initial vascular damage and subsequent reparative changes within the spinal cord appear to adequately explain the cavitation observed
Hall, P. V. 1975	Syringomyelia (Dog)	15	Exploration of the pathogenesis of hydrosyringomyelia, ischemic myelopathy, and syringomyelia following adhesive arachnoiditis	C: No operation (*n* = 2) I1: Kaolin (250 mg) + cerebrospinal fluid (1 mL) in intracisternal space (*n* = 10) I2: Rhizotomies at T6 in bilateral epidural space + Laminectomy at T9‐L3 (*n* = 3)	Cavities in the spinal cord of dogs appearing after the intracisternal administration of kaolin are not due to ischemic softening, but rather to distention and subsequent rupture of the central canal. Ischemic lesions could be produced and were found to have quite different histopathology; the hydrodynamic hypothesis of cavitary myelopathy following adhesive arachnoiditis is a compensatory mechanism to the associated hydrocephalus

Abbreviations: 3D‐CISS, Three dimensional‐constructive interference in steady state magnetic resonance imaging; AA, Adhesive arachnoiditis; C, control group; CSF, Cerebrospinal fluid; EF, Epidural fibrosis; GF, Gelfoam; Gy, Gray unit; HA: Hyaluronic acid; I, intervention group; MMC, Mitomycin C; MRI, Magnetic resonance imaging; P407, Poloxamer 407; PTS, Posttraumatic syringomyelia; QA, Quisqualic acid; Ro5‐4864, A peripheral benzodiazepine receptor agonist; Rt‐PA: Recombinant tissue plasminogen activator; SAA, Spinal adhesive arachnoiditis; SAD, Spinal arachnoid diverticula; SCI, Spinal cord injury; TAM, Tamoxifen; TGF‐β1, Transforming growth factor beta 1.

^a^ Compared with the control group.

## 4 Discussion

SAA, previously considered a rare disease, has been increasing among elderly patients undergoing MRI of the spine. However, the clinical awareness of SAA is still very unsatisfactory. Most published studies related to SAA were limited to either case reports or case series reports. This study was the first comprehensive review of both clinical and animal model investigations of SAA.

### 4.1 General Clinical Features of SAA

#### 4.1.1 Common Symptoms of SAA

Through this systematic review, we demonstrated that the mid‐aged population (mean age 44.2 ± 17.53 years) with a long diagnostic window of 12 [3, 4, 5, 6, 7, 8, 9, 10, 11, 12, 13, 14, 15, 16, 17, 18, 19, 20, 21, 22, 23, 24, 25, 26, 27, 28, 29, 30, 31, 32, 33, 34, 35, 36] months could be at higher risk of developing SAA symptoms. Most patients suffered from SAA for the first time (75.00%), while other patients had recurrent SAA (25.00%). Progressive neurological deterioration was seen in nearly 50% of patients if no intervention was conducted. The most common symptoms of SAA were abnormal motor function (78.75%), abnormal nerve sensations in related dermatomes (39.58%), and pain (37.50%), which might be because of the spinal cord or nerve roots failing to manage the input or output signals from the peripheral nervous system or central nervous system (CNS) in these patients.

More specifically, symptoms of motor dysfunction were likely to present different degrees of paralysis (36.77%), gait disturbances (24.87%), bladder incontinence (24.34%), weakness (23.81%), and fecal incontinence (15.34%). The pain symptoms were mainly in the lower back (44.33%), sciatica (17.22%), and leg (12.22%) regions. Patients complained of paresthesia (e.g., numbness and tingling sensations) (47.89%), and dysesthesia (e.g., burning and electrical sensations) (20.53%) as the most obvious manifestations of abnormal sensory functions, while others encountered different degrees of sensation loss, such as hypoesthesia (26.32%) and even complete loss of sensation (5.26%).

#### 4.1.2 Imaging Analysis of SAA Patients

Since SAA usually coexists with other degenerative spinal diseases, MRI was used as the primary screening imaging method in most cases. Myelography used to be the gold standard for the diagnosis of AA in earlier times; however, it is now only conducted in a small percentage of patients (25.00%) due to its invasive nature and potential risks of allergy and infection. We also found that the injection of contrast during myelography could also be a possible risk factor for SAA. Only a few cases underwent diagnostic surgery, post‐surgery histopathological examination, and fiberscope. Moreover, the sensitivity and specificity of MRI in diagnosing SAA were relatively higher (92% and 100%) [1, 43]. Reasonably, MRI examination is now becoming the mainstream method for the confirmatory diagnosis of SAA.

Recently, high‐resolution MRI and SAA‐specific sequences designed for detecting CSF obstruction have been introduced to diagnose SAA. T‐SLIP MRI is also reportedly valuable for revealing fluid communication between two CSF‐containing ventral spaces [44]. 3D‐CISS MRI could provide a high contrast‐to‐noise ratio, facilitating to distinguish arachnoid cysts from other pathological changes associated with abnormal CSF dynamics [45, 46, 47]. The indirect evidence of CSF flow impairment in MRI imaging could help physicians differentiate the diagnosis of SAA without applying any invasive procedures.

#### 4.1.3 SAA Lesions in Different Spinal Locations

More than 60% of SAA patients presented an extensive form of lesion, involving more than two vertebral segments. Arachnoid membranes in the thoracic and lumbar vertebral regions had higher incidences of SAA than that in the cervical and sacrococcygeal vertebral regions. This might be because of spinal trauma, spinal surgery, or epidural anesthesia, usually located in the thoracic and lumbar vertebral regions.

Intriguingly, SAA in the cervical region is more susceptible to spreading to its adjacent thoracic region. We found that most lesions occurred in the lower cervical and upper thoracic regions, which could be explained by the anatomical studies of spinal arachnoid space, since the posterior subarachnoid space in this region contains numerous arachnoid strands and fibrils [48, 49]. These anatomical structures predispose this region to inflammation and/or infectious diseases, leading to arachnoidopathy. Moreover, arachnoid recesses in this region are surrounded by small lymphatic vessels that connect with satellite lymph nodes [48, 50, 51]. As most lymph nodes are in the cervicothoracic area, we presumed that inflammatory responses in this area would be more prominent than in other vertebral regions.

Additionally, we evaluated whether SAA in different vertebral segments would have differential etiologies and found that thoracic SAA might have been caused by the mechanical injury of trauma/spinal surgery, while cervical SAA might have originated from meningitis‐associated inflammatory damages.

### 4.2 Treatment and Prognosis of SAA

Most patients preferred surgery to treat SAA (359/392, 91.58%), while a few patients chose conservative interventions (33/392, 8.42%). This might be because SAA often combines with other degenerative spinal diseases, which also need surgical interventions. The overall post‐surgery recovery rate (84.12%) was significantly higher than that for the conservative interventions (51.52%). The SAA patients with cervical lesions had the highest post‐surgery recovery rate compared with that in the thoracic, lumbar, or sacrococcygeal region.

However, this result should be interpreted with caution. First, there was no standardized guideline in the conservative treatment (epidural steroid injection was mostly given), which led to a small sample size in prognostic data. Second, selection bias was widely noticed in case reports and case series of post‐surgery AA patients. We found that spinal surgery was one of the major etiologies of SAA. Moreover, minor AA is prone to be self‐limited [52]. Therefore, it is vital to find a balance between choosing either conservative treatment or removal surgery.

In animal studies of new therapeutics targeting AA, Keskin et al. [25] have found that Ro5‐4864 could be effective in reducing fibrosis and arachnoid involvement. Ozturk et al. [26] have shown that TAM may be effective in reducing fibrosis and arachnoid involvement. Cemil et al. [23] have demonstrated that the application of local pimecrolimus or MMC could effectively reduce epidural fibrosis and adherence. Current drug research has revealed that substances such as Ro5‐4864 and TAM possess the ability to suppress fibroblast proliferation and collagen fiber secretion by targeting transforming growth factor‐beta 1 (TGF‐β1). This discovery undoubtedly presents a promising approach for the conservative management of SAA.

### 4.3 The Pathogenesis of SAA

We observed that spinal trauma/surgery, infectious meningitis, spinal subdural hemorrhage, Chiari malformation type I, and epidural anesthesia were the most common causes of SAA pathogenesis. Minor causes of SAA included idiopathic AA, familial AA, and autoimmune disease or spinal bifida.

#### 4.3.1 SAA and Degenerative Spinal Diseases

**FIGURE 2:**
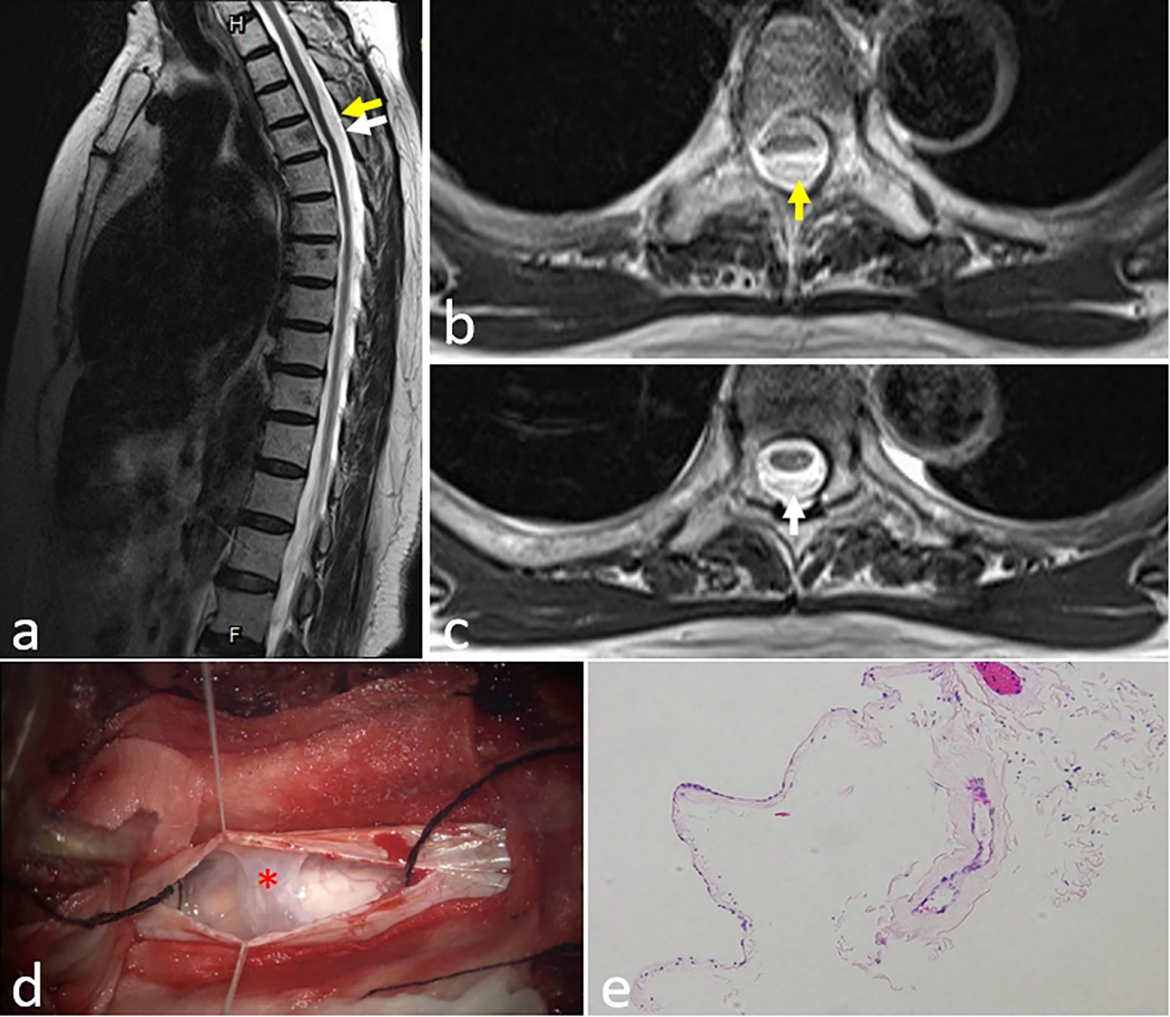
Case presentation of a patient with extensive adhesive arachnoid combined with secondary arachnoid cysts. A 70‐year‐old female presented with neck and lower back pain for 6 months. The MRI imaging showed multiple sites of adhesive arachnoid and arachnoid cysts at T3 and T4 levels. During surgery of adhesive arachnoid lysis and arachnoid cyst resection, specimens were taken for pathological examinations. (a) Sagittal and (b, c) axial T2‐weighted spine MRI demonstrated arachnoid cysts in T3 and T4 levels (yellow arrow) and extensive adhesive arachnoid (white arrow); (d) intraoperative view to the sites of adhesive arachnoid (red asterisk); (e) histopathological examination of the specimen showed thickening and fibrosis of the arachnoid (H&E staining, 20× magnification).

**FIGURE 3:**
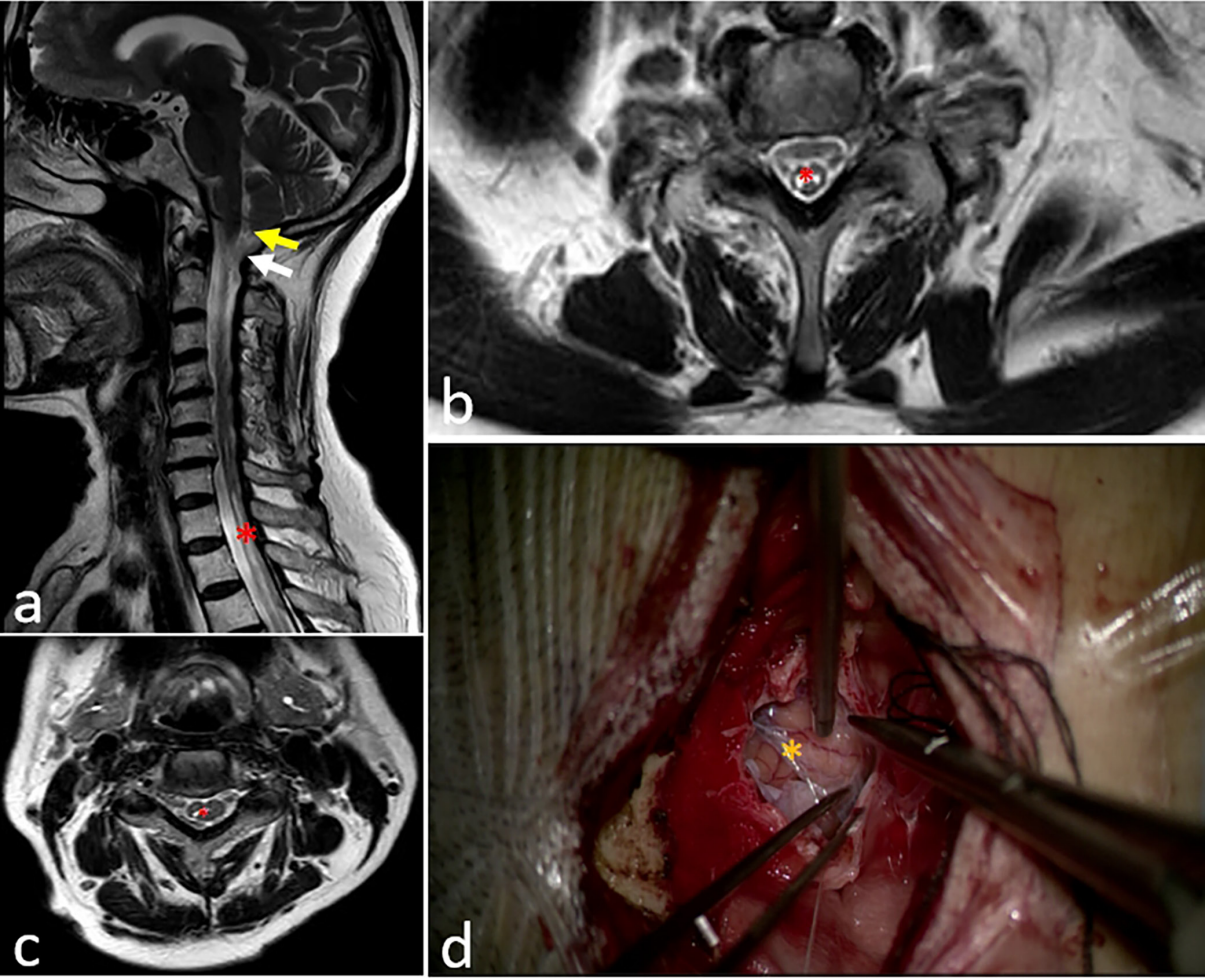
Case presentation of a patient with extensive adhesive arachnoid combined with secondary syringomyelia. A 76‐year‐old female presented with progressive neck pain, weakness, and numbness in her right upper limb for 2 years. The MRI imaging exhibited Chiari malformation type I and extensive adhesive arachnoid with secondary syringomyelia. Foramen magnum decompression was performed. (a) Sagittal and (b, c) axial T2‐weighted spine MRI demonstrating Chiari malformation (yellow arrow) and extensive adhesive arachnoid (white arrow) and syringomyelia (red asterisk); (d) intraoperative view to the sites of adhesive arachnoid (orange asterisk).

The prevalence of DSDs over the entire spine was 71% in men and 77% in women aged < 50 years; however, in older adults (> 50 years), the prevalence of DSD reached over 90% irrespective of the gender difference [53]. As the vertebral disk degenerates, it may initiate herniation and develop bone spurs, which then occupy extra spaces pressing the spinal cord and surrounding nerves to one side of the canal. The compression of the nerves and spinal cord onsets pain, muscle weakness, or numbness in certain areas depending on the segment affected by disc degeneration. In our clinical practice, we found that AA membranes coexisted with degenerative spinal lesions during spine surgery in DSD patients (Figures 2 and 3). Several studies have also reported similar findings. Crisi et al. have found that 30% of SAA patients had ipsilateral intradural root enhancement from the site of compression to the medullary root junction [54]. Jackson and Isherwood [55] have demonstrated that nerve root aggregation and clumping caused by spinal stenosis could be associated with limited AA.

The underlying mechanism of the crosstalk between SAA and DSD remains unclear. The intervertebral disk is the largest avascular organ in the body and is considered the site of immune privilege [56]. The rupture of the posterior longitudinal ligament, fibrous ring, and protrusion of the nucleus pulposus can potentially trigger the development of autoimmunity, eventually inducing inflammatory responses in the arachnoid membrane [57, 58, 59].

However, the symptoms of some patients with intervertebral disk herniation and spinal stenosis were not improved even after vertebral fusion surgery. This might be because the spinal surgery itself causes direct mechanical injury to arachnoid membranes, thereby exacerbating inflammatory responses around the lesion area. We also found that mechanical injuries to arachnoid membranes such as trauma (22.70%), surgery (15.37%), anesthesia procedures surrounding the spinal region (e.g., epidural anesthesia, spinal anesthesia, and paravertebral anesthesia) (5.44%), Chiari malformation type I (5.67%), spinal injections (e.g., intrathecal chemotherapy or epidural steroid injection) (3.07%), spinal deformities, birth trauma, recurrent lumbar puncture (4.49%) caused more than 50% of SAA.

#### 4.3.2 SAA and Infectious Meningitis

Infectious meningitis, most likely tuberculous meningitis (TBM), was the second major cause of SAA (17.73%). TBM usually presents as chronic relapsing meningitis [60]. But chronic meningitis due to other pathogens, such as staphylococcus, listeria monocytogenes, diplococci, neurocysticercosis, sparganosis, cryptococcus, coccidioidomycosis, herpes simplex virus (HSV), and varicella‐Zoster virus (VZV), was rarely reported [61, 62, 63, 64, 65, 66, 67, 68]. Long‐term inflammation of the arachnoid and pia mater increases the collagen deposition between the two layers, leading to adhesions [69, 70]. Radicular nerve roots in the related area become edematous and hyperemic, and then get entrapped and clumped together in the adhesive leptomeninges. The nerve function is eventually compromised due to diminished blood supply [71].

#### 4.3.3 SAA and Cerebral Hemorrhage

Hemorrhage is another main cause of SAA, accounting for more than 10% of SAA cases (13.48%), although SAA is a complication of cerebral hemorrhage in less than 1% of cases [72, 73, 74]. The major form of hemorrhage included spinal subarachnoid hemorrhage (91.23%), subdural hemorrhage (7.02%), and epidural hematoma (1.75%). Several case reports presented similar clinical scenarios in which post‐hemorrhagic patients suffered from progressive spinal cord compression symptoms due to extensive arachnoiditis [75]. Microscopic examination of these specimens indicated that disclosed particles of hemosiderin or lysed erythrocytes in dense connective tissues might contribute to a leptomeningeal inflammatory reaction [76, 77]. Moreover, massive hemorrhage could contaminate the spinal subarachnoid space, especially the cervical and thoracic regions. This might be because prolonged decubitus in patients with severe cerebral hemorrhage could cause stagnation of blood, disturbing the CSF circulation and accumulation of inflammatory biomarkers.

#### 4.3.4 SAA and Other Minor Causes

SAA was formerly considered a sporadic condition with common etiologies, such as spinal surgery and inflammation or infection. However, in our clinical practice, we were curious about why some patients were predisposed to develop SAA while others were not. Therefore, we summarized the cases either with no clear cause or genetic cause of SAA. Both idiopathic and familial SAA accounted for 5% of all SAA cases. Reports about familial cases of arachnoiditis are only limited to the articles of Duke and Hashimoto and Nagai et al., which are of Japanese origin. The other article by Pasoglou et al. was of Belgian origin [78, 79, 80]. They demonstrated that SAA might be very strongly influenced by a very rare inherited genetic anomaly with incomplete penetrance of a major gene. Multigenic inheritance is also a possibility. Further genetic studies are needed in the future.

### 4.4 Multiple Pathological Forms of SAA and Related Complications

SAA exhibits various pathological forms. AA is just one of the various pathological forms of arachnoiditis. The other common forms include AA combined with arachnoid cyst (Figure 2), arachnoid calcification/scars, and arachnoid web/fibrosis. More than 30% of SAA patients develop secondary syringomyelia. Only rare cases of arteriovenous fistula secondary to SAA have been reported (*n* = 5) [81, 82, 83, 84].

#### 4.4.1 Multiple Pathological Forms of SAA

The common pathological diagnosis of SAA was AA (80.82%), AA combined with arachnoid cyst (12.79%), arachnoid calcification/scars (3.43%), and arachnoid web/fibrosis (2.97%).

##### 4.4.1.1 AA Combined With Arachnoid Cyst

The underlying pathomechanism of AA‐induced arachnoid cysts formation is poorly understood. Under the action of a series of inflammatory processes, the fibrosis of the subarachnoid space results in the formation of arachnoid trabeculae. Inflammation compartments and proliferates, leading to scarring and fibrosis, and secondary thickening of the arachnoid, thereby impeding the normal flow of CSF in the arachnoid space, which then creates isolated areas inducing the formation of cystic lesions [66, 85, 86]. The subsequent enlargement of cysts may be attributed to mechanisms such as a one‐way valve mechanism, intracystic osmosis, and/or secretion of cells from the cyst wall [66, 87]. The spinal cord exhibits distinct anatomical characteristics between the dorsal and ventral regions. In cases of hemorrhage or traumatic disorders affecting the spinal subarachnoid space, the dorsal subarachnoid space is particularly susceptible to adhesions due to its abundance of arachnoid trabeculae and veins [86]. While ventral arachnoid cysts rarely occur. Haimoto, Nishimura, and Ginsberg [88] suggest that ventral thoracic intradural spinal arachnoid cysts (ISAC) have a higher propensity to develop due to their extensive craniocaudal extension and presence of intracystic fibrous septae. Additionally, the formation of multiple cysts may not transpire concurrently. Gutierrez et al. [89] have reported that arachnoiditis progresses at various stages along the nerve axis, and the development of arachnoid cysts necessitates sufficient arachnoid thickening, adhesions, and CSF flow. Cysts in the upper segment may mature earlier, impeding the downward flow of CSF and subsequently restricting the formation of cysts in the lower segment.

##### 4.4.1.2 Arachnoid Ossificans

Arachnoid ossificans is the end‐stage manifestation of chronic AA. Brunner et al. [90] have found yellow bone marrow in the bone cavity of arachnoiditis ossificans, which they believe to be caused by arachnoid fat metaplasia. Persistent inflammatory responses can also lead to chronic fibroblastic proliferation of the pia mater with overproduction of collagen tissue and intradural scarring. Subsequently, osteoblast proliferation and ossification increase, leading to progressive ossification [12, 90, 91, 92]. Systemic metabolic conditions, particularly alterations in calcium metabolism, such as hyperparathyroidism, appear to predispose to arachnoiditis ossification [90].

#### 4.4.2 Common Complications of SAA

##### 4.4.2.1 Secondary Syringomyelia and SAA‐Related CSF Circulation Disturbances

Chronic AA may cause disturbances in the CSF circulation, which can play a major role in the formation of secondary syringomyelia. Chang and Nakagawa [93] have established an electrical circuit model of CSF dynamics in the spine and analyzed the formation mechanism of AA leading to cavities. They believe that arachnoid adhesions would lead to CSF circulation disorder. Because the CSF does not circulate below the adhesion site, the pressure in the subarachnoid space decreases, and the amount of circulating CSF through the central canal below the adhesion site increases. The pressure difference between the central canal and the subarachnoid space is formed, resulting in the continuous expansion of the spinal cord, eventually facilitating the formation of syringomyelia. Kobayashi et al. [36] suggest that the disturbance of CSF flow can be closely related to the onset of hydrospinal fluid. The onset of syringomyelia appears to be related to disruption of the blood–spinal cord barrier caused by intramedullary blood flow disturbances, particularly venous congestion, resulting in intramedullary edema. The intramedullary edema then leads to necrosis of the nerve tissue. This state of swelling is equivalent to a pre‐syringomyelia state [94, 95, 96]. When a necrotic tissue is phagocytized by macrophages, a cavity will form.

AA can also lead to some other types of complications, such as arteriovenous fistula. When the adhesion was located in the lumbosacral area, it leads to the development of the filum terminale arteriovenous fistula (FTAVF). Long‐term severe lumbar stenosis may cause repetitive microtrauma, leading to hyalinization with fibrosis and development of AA, and eventually the increased tension of filum terminale and arteriovenous fistula [81, 82, 83, 84].

## 5 Conclusions

SAA is caused by arachnoid inflammatory reactions. Surgery, trauma, bleeding, and infection are the most common causes of SAA, which could cause a series of atypical clinical manifestations such as pain, movement disorders, and sensory disturbances. The pathological consequences of SAA are more complex than AA, and manifest in different forms, such as AA combined with arachnoid cyst, arachnoid calcification/scars, and arachnoid web/fibrosis. In many instances, AA was associated with secondary syringomyelia. Owing to the absence of established clinical diagnostic and treatment standards for AA, misdiagnosis or failure to diagnose the actual pathologic condition can occur. Therefore, accurate diagnosis necessitates a comprehensive assessment comprising of medical history, MRI, and other relevant imaging examinations. Considering the increasing incidence rate of SAA, it is imperative to allocate more attention and clinical effort to this condition. Further in‐depth research on SAA is required to provide valuable guidance for the clinical management of the disease.

## Author Contributions

W.Z. and Z.L. finished the study and wrote this paper. W.Z., Z.L., K.W., and L.Z. made data collection and analysis. W.Z., Z.L., S.L., X.Z., Y.W., and K.H. revised the manuscript and edited English. H.W. contributed to the conception and design of this study and proposed the amendments. W.Z., H.W., and Z.L. took full responsibility for the data, the analyses and interpretation, and the conduct of the research.

## Ethics Statement

The authors have nothing to report.

## Consent

The authors have nothing to report.

## Conflicts of Interest

The authors declare no conflicts of interest.

## Supporting information


Table S1

## Data Availability

The data and materials related to the current study are available from the corresponding author on reasonable request.
