# Supplementary material for: Spinal Adhesive Arachnoidopathy, the Disorder More Than Simply Adhesive Arachnoiditis: A Comprehensive Systematic Review of 510 Cases
Source: CNS Neurosci Ther. 2024 Oct 22;30(10):e70084. doi: 10.1111/cns.70084 (PMC11494685; doi:10.1111/cns.70084)
Supplement: Supplementary file 1 — Table S1 [file CNS-30-e70084-s001.docx]

**Supplementary Table. 1 Search Strategies**

Search included: PubMed Central (PMC), EMBASE, and Cochrane Library: search date was from the inception through October 2022

**1) PubMed Central (PMC) search strategy**

| 1. adhesive arachnoiditis[Title/Abstract] |
| --- |
| 1. adhesive leptomeningitis[Title/Abstract] |
| 1. arachnoid adhesion[Title/Abstract] |
| 1. arachnoid calcification[Title/Abstract] |
| 1. arachnoid ossificans[Title/Abstract] |
| 1 OR 2 OR 3 OR 4 OR 5 |

**2) Embase search strategy**

| 1. adhesive arachnoiditis:ab,ti,kw |
| --- |
| 1. adhesive leptomeningitis:ab,ti,kw |
| 1. arachnoid adhesion:ab,ti,kw |
| 1. arachnoid calcification:ab,ti,kw |
| 1. arachnoid ossificans:ab,ti,kw |
| 1 OR 2 OR 3 OR 4 OR 5 |

**2) Cochrane Library search strategy**

| 1. adhesive arachnoiditis:ab,ti,kw |
| --- |
| 1. adhesive leptomeningitis:ab,ti,kw |
| 1. arachnoid adhesion:ab,ti,kw |
| 1. arachnoid calcification:ab,ti,kw |
| 1. arachnoid ossificans:ab,ti,kw |
| 1 OR 2 OR 3 OR 4 OR 5 |
